# Research progress of treating hyperuricemia in rats and mice with traditional Chinese medicine

**DOI:** 10.3389/fphar.2024.1428558

**Published:** 2024-07-19

**Authors:** Haodong Bai, Zidong Zhang, Mingtao Zhu, Yanping Sun, Yimeng Wang, Biao Li, Qiuhong Wang, Haixue Kuang

**Affiliations:** ^1^ Key Laboratory of Basic and Application Research of Beiyao, Heilongjiang University of Chinese Medicine, Ministry of Education, Harbin, China; ^2^ School of Life Sciences, Guangzhou University, Guangzhou, China

**Keywords:** hyperuricemia, Chinese herbal medicine, Chinese herbal formulae, mechanism of action, urate transporter

## Abstract

Hyperuricemia (HUA) is a common chronic metabolic disease caused by abnormal purine metabolism and uric acid excretion. Despite extensive research on HUA, no clear treatment has been found so far. Improving purine metabolism and promoting uric acid excretion is crucial for the effective treatment of HUA. In recent years, traditional Chinese medicine and traditional Chinese medicine prescriptions have shown good effects in treating HUA. This article summarizes the latest progress in treating HUA in rats and mice using traditional Chinese medicine and prescriptions, elaborates on the pathogenesis of HUA, explores the application of commonly used traditional Chinese medicine treatment methods and prescriptions, and discusses the previous pharmacological mechanisms. In general, our research indicates that traditional Chinese medicine can effectively relieve the symptoms related to elevated uric acid levels in HUA rats and mice. However, further exploration and research are needed to verify its efficacy, safety, and feasibility.

## 1 Introduction

**FIGURE 1:**
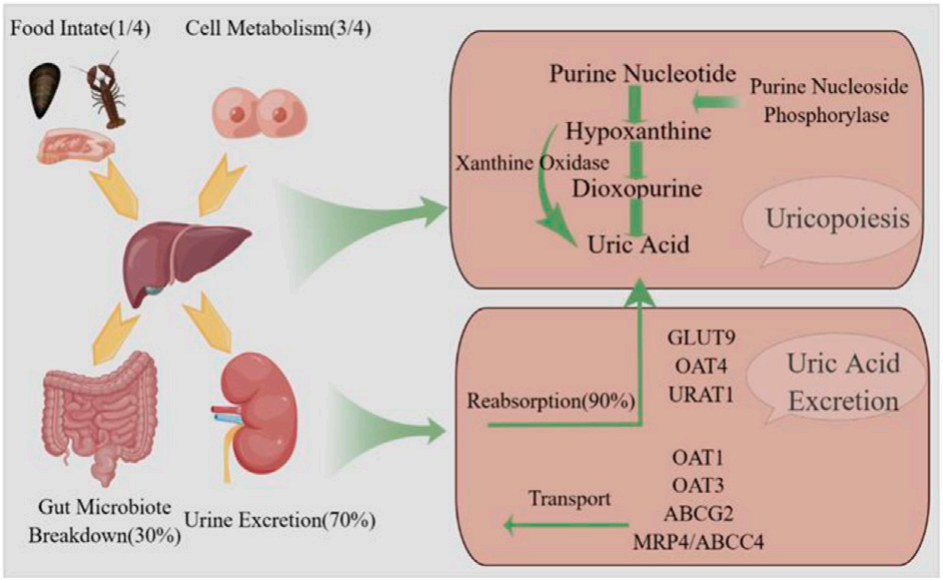
Uric acid production and excretion pathway. Uric acid is synthesized in the liver via the enzymatic action of xanthine oxidase and subsequently eliminated from the body through both renal.

Hyperuricemia is a prevalent chronic metabolic disorder resulting from impaired purine metabolism and inadequate uric acid excretion (Wang et al., 2022). The process of uric acid production and excretion is shown specifically in Figure 1. In males, HUA is generally diagnosed when the serum uric acid concentration exceeds 420 μmol/L, while in females, the threshold is set at 360 μmol/L (Johnson et al., 2018). The global prevalence of HUA has increased due to improvements in living standards and dietary patterns. A nationally representative cross-sectional survey conducted during 2018–2019 estimated that approximately 14% of Chinese adults were affected by HUA (Zhang et al., 2022). HUA can contribute to the development of gout, kidney disease, type 2 diabetes mellitus, as well as cardiovascular and cerebrovascular disorders, and Intestinal disorders, significantly impacting individuals’ overall wellbeing and health status (Yanai et al., 2021).

At present, lifestyle improvement and drug therapy are the main means to control uric acid levels. Studies have confirmed that quitting smoking and drinking, a low-purine diet, and reducing the intake of greasy and high-fat foods can effectively reduce hyperuricemia (Morgan and Singh, 2021). Extensive research has been conducted on this disease and some drugs, such as benzbromarone, sulfinpyrazone, and propoxur have been utilized in its treatment (Strilchuk et al., 2019). Although these drugs demonstrate positive effects in reducing uric acid levels, prolonged usage can lead to adverse effects including skin rashes, liver damage, and potentially severe kidney complications (Bose et al., 2014; Yang et al., 2022). Therefore, there is a need to develop medicines or natural medicinal ingredients that are safer and more effective.

Compared with traditional drugs for the treatment of high uric acid, the development of herbal extracts (e.g., aqueous extracts, alcoholic extracts, and active metabolites), which are characterized by multi-links, multi-levels, multi-targets, and low toxicity and side-effects, has become a new idea for the development of uric acid-reducing drugs. In recent years, more and more studies have been devoted to the mining of natural substances with uric acid-reducing activity from herbs. Studies have shown that plant metabolites, such as flavonoids, saponins, polysaccharides, and polyphenols, regulate uric acid metabolism by inhibiting the activity of key enzymes of uric acid synthesis and regulating various pathways such as uric acid transporter proteins, thus preventing or treating hyperuricemia and its complications. For example, both the aqueous and alcoholic extracts of Agrocybe aegerita were able to exhibit inhibitory effects on hepatic xanthine oxidase (XOD) activity and elevate renal organic anion transporter protein 1 (OAT1) in hyperuricemic mice (Yong et al., 2018). Er Ding Granules (EDG) have been shown to effectively lower uric acid levels by down-regulating glucose transporter protein 9 (GLUT9) and urate anion transporter protein 1 (URAT1), while up-regulating organic anion transporter 1 (OAT1). These actions enhance uric acid excretion and reduce its production, providing a balanced and sustained therapeutic effect. EDG’s multi-target approach offers potential advantages over traditional urate-lowering drugs such as allopurinol and febuxostat, potentially leading to fewer side effects in the management of hyperuricemia (Zhang W. et al., 2019). Compared to conventional methods, traditional Chinese medicine (TCM) offers several advantages in the treatment of hyperuricemia (HUA). One major advantage is TCM’s multi-target, multi-component approach, which can simultaneously address various pathways of uric acid metabolism. This comprehensive approach not only helps reduce serum uric acid levels but also alleviates related complications such as gout, hypertension, diabetes, and chronic kidney disease, contributing to overall health (Yang et al., 2022). To summarize, this paper collects various related literature published in recent years from PubMed, Web of Science, CNKI, and other electronic databases with “hyperuricemia, traditional Chinese medicine, botanical medicine, drug metabolite, traditional Chinese medicine compound” as the main keywords, to provide a theoretical basis for the research and development of uric acid-lowering drugs. To provide a theoretical basis for the research and development of uric acid-lowering drugs. All botanical drugs’ names have been checked with Plants of the Word Online (http://www.plantsoftheworldonline.org).

## 2 Pathogenesis of hyperuricemia

The pathogenesis of hyperuricemia is complex. A growing body of research now suggests that the pathogenesis of hyperuricemia may be related to enzyme activity, uric acid transporter proteins, intestinal flora dysbiosis, and oxidative stress and inflammatory responses.

### 2.1 Dysregulation of enzyme activity

Uric acid synthesis involves a variety of enzymes, including xanthine XOD as the key enzyme in the body of uric acid production, purine nucleoside phosphorylase PNP catalyzed creatinine catabolism to produce hypoxanthine to further catalyze the oxidation of intermediate products xanthine, and ultimately xanthine oxidation into uric acid. When XOD activity is dysregulated, the higher activity of XOD will accelerate the catalytic oxidation of hypoxanthine and xanthine, resulting in a continuous increase in uric acid levels and hyperuricemia (Chen et al., 2023). XOD catalyzes the oxidation and hydroxylation of hypoxanthine and xanthine to uric acid, generating reactive oxygen species such as superoxide anion and hydrogen peroxide at the flavin center. Elevated levels of these reactive oxygen species can lead to oxidative stress and ischemia-reperfusion injury. This oxidative stress can contribute to conditions such as hyperuricemia and metabolic syndrome (Borges et al., 2002).

### 2.2 Imbalanced expression of uric acid transporter protein

Uric acid in the body is mainly excreted through the kidneys (about 2/3), and a small portion is excreted through the intestines (about 1/3), the uric acid transporter protein in the kidneys is the regulation of the dynamic balance of uric acid reabsorption and excretion, and plays an important role in the process of uric acid excretion (Maiuolo et al., 2016). Uric acid transporter proteins are mainly classified into two groups, one is reabsorption transporter proteins, mainly including urate anion transporter protein 1 (URAT1), organic anion transporter protein 4 (OAT4) and glucose transporter protein 9 (GLUT9); the other is secretion-associated transporter proteins, mainly including organic anion transporters 1 and 3 (OAT1 and OAT3), multidrug resistance protein 4 (MRP4/ABCC4) and ATP-binding cassette subfamily G member 2 (ABCG2). Over-expression of uric acid reabsorption transporter proteins leads to abnormal uric acid reabsorption, resulting in elevated serum uric acid levels; under-expression of uric acid secretion transporter proteins causes a decrease in renal uric acid secretion and insufficient excretion results in elevated serum uric acid levels. The literature has reported that 90% of patients with hyperuricemia have an imbalance between renal excretion and expression of uric acid transporter protein (Russo et al., 2022).

### 2.3 Oxidative stress and inflammatory response

Uric acid production is accompanied by XOD, which promotes the activation of reduced coenzyme Ⅱ and the release of reactive oxygen species. When uric acid levels are higher than normal physiological levels, oxidative stress damage to the body is amplified (Kurajoh et al., 2021). Oxidative stress *in vivo* can activate related inflammatory factors and inflammatory pathways and induce innate immune responses, while the activation of related pro-inflammatory factors can induce inflammation. It has been reported that the development of hyperuricemia is closely related to oxidative stress and inflammatory responses (Liu et al., 2021).

### 2.4 Imbalance of intestinal homeostasis

Transporter proteins that promote uric acid excretion are also present in the gut, and these transporter proteins are involved in the metabolic processes of gut microorganisms (Hosomi et al., 2012). ABCG2, which is distributed in different parts of the small and large intestine, is a major uric acid secretion transporter protein that dominates intestinal uric acid excretion and regulates blood uric acid levels. Studies have shown that increased expression of ABCG2 was found in the intestine of denervated rats with impaired renal excretion, suggesting that increased expression of this transporter protein may be the key to intestinal excretion of uric acid (Bhatnagar et al., 2016). In addition, chronic inflammation is a typical pathological feature of hyperuricemia (Zhou et al., 2018). Intestinal flora may ameliorate hyperuricemia by repairing the intestinal mucosal barrier and attenuating the inflammatory response. Elevated levels of inflammatory factors negatively affect both epithelial integrity of the gut and gut flora homeostasis (Luissint et al., 2016). Dysbiosis of the intestinal flora increases intestinal permeability and promotes translocation of bacteria or bacterial products such as lipopolysaccharide (LPS) (Xu et al., 2019). Elevated serum LPS levels induce chronic inflammation and increase the risk of developing hyperuricemia. In addition, LPS is a metabolite of intestinal flora, and abnormal levels of LPS in the circulation are usually accompanied by an increase in the activity of XOD, an important enzyme in the oxidative metabolism of purines. Thus, dysbiosis of the intestinal flora and impaired intestinal barrier repair can lead to elevated levels of LPS in the circulation, causing chronic inflammation, which is a new factor in the pathogenesis of hyperuricemia.

## 3 Uric acid-lowering effects of botanical drugs extracts, active metabolites, and herbal formulas in hyperuricemic rats and mice

**FIGURE 2:**
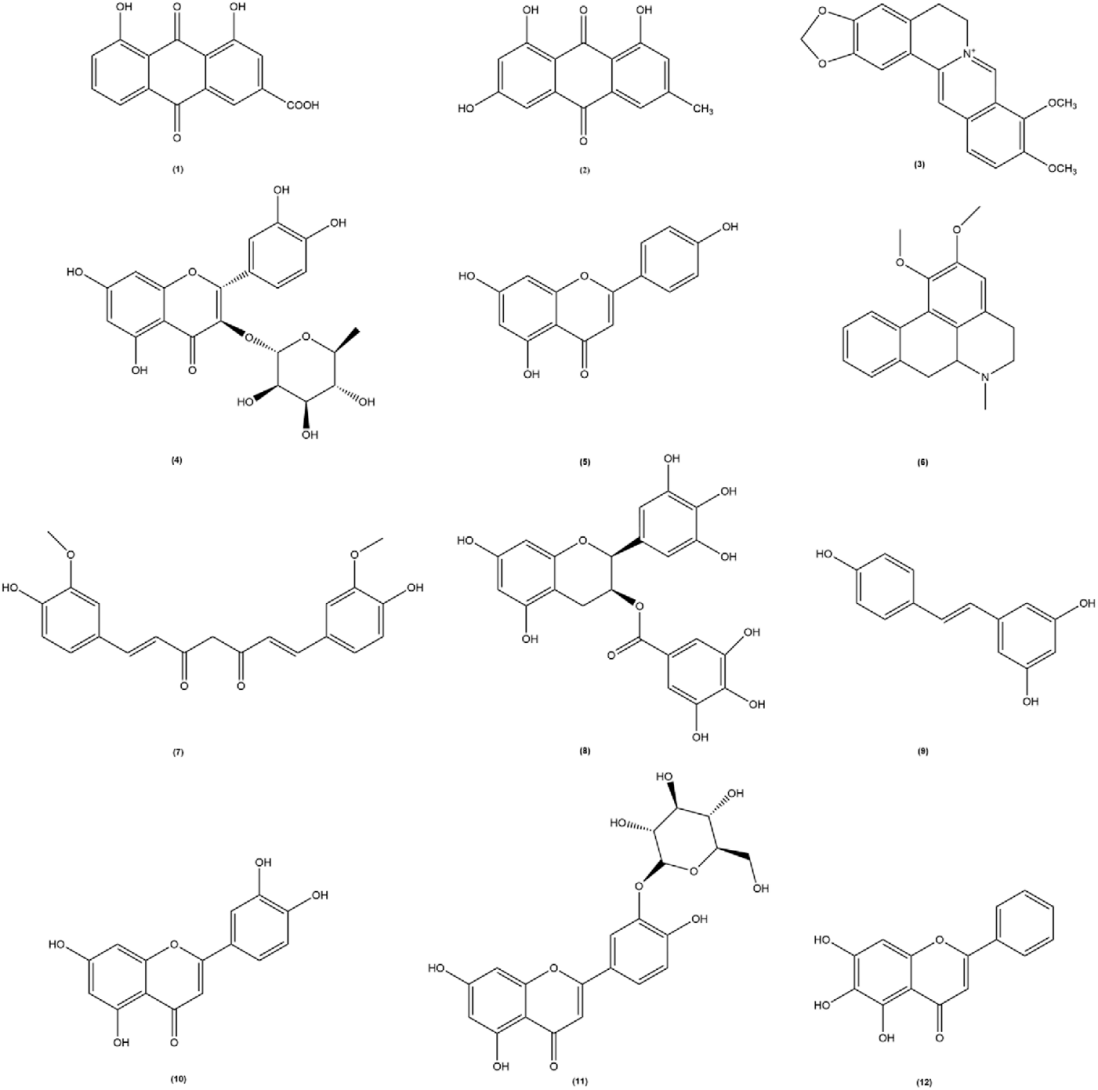
The chemical structures of the metabolites involved in the thesis. Among them, (1) Rhubarbic Acid (Molecular formula: C_15_H_8_O_6_), (2) Emodin (Molecular formula: C_15_H_10_O_5_), (3) Berberine (Molecular formula: C_20_H_18_NO_4_^+^), (4) Astilbin (Molecular formula: C_21_H_22_O_11_), (5) Apigenin (Molecular formula: C_15_H_10_O_5_), (6) Nuciferine (Molecular formula: C_19_H_21_NO_2_), (7) Curcumin (Molecular formula: C_21_H_20_O_6_), (8) Epigallocatechin-3-gallate (Molecular formula: C_22_H_18_O_11_), (9) Resveratrol (Molecular formula: C_14_H_12_O_3_), (10) Luteolin (Molecular formula: C_15_H_10_O_6_), (11) Luteolin-4′-O-glucoside (Molecular formula: C_21_H_20_O_11_), (12) Baicalein (Molecular formula: C_15_H_10_O_5_).

In recent years pharmacological studies have validated the therapeutic effects of several botanical drug extracts, active metabolites, and herbal formulas by establishing hyperuricemic animals. The respective uric acid-lowering effects and the potential mechanisms of action are summarized in Tables 1–3, respectively. The chemical structures of the metabolites described in the paper are shown in Figure 2.

**TABLE 1:** Mechanism of action of botanical drugs extracts in preventing HUA.

Single botanical drugs extracts	Dosage	Object	Effect/Mechanism	Controls	References
*Astragalus membranaceus* Bunge [Fabaceae; *Astragali* radix] extract	0.25, 0.5, 1 g/kg 6 w	HUA mice (*in vivo*)	Upregulation of ABCG2, downregulation of URAT1 and GLUT9, protection of the intestinal mucosal barrier, inhibition of renal inflammation	Benzbromarone group (20 mg/kg positive control)	Wang et al. (2023a)
*Atractylodes macrocephala* Koidz. [Asteraceae; *Atractylodis* rhizome] extract	50, 100, 200 mg/kg 42 d	HUA rat (*in vivo*)	Inhibition of XOD activity, activation of AMPK/SIRT1 signaling pathway, inhibition of NF-κB activation, inhibition of macrophage polarization	Allopurinol group (27 mg/kg positive control)	Qian et al. (2023)
*Cichorium glandulosum* Boiss. & A.Huet [Asteraceae; *Cichorii herba cichorii* radix] extract	6.6, 13.3, 16.7 g/kg 60 d	HUA quail (*in vivo*)	Inhibition of XOD activity, Inhibition of renal TLR4/NF-κB inflammatory pathway, protection of intestinal mucosal barrier	Benzbromarone group (20 mg/kg positive control)	Bian et al. (2020)
*Plantago asiatica* L. [Plantaginaceae; Plantaginis Semen] extract	0.9375, 1.875, 3.75 g/kg 28 d	HUA rat (*in vivo*)	Downregulation of URAT1, inhibition of PI3K/Akt inflammatory pathway regulation of lipid metabolism disorder	Benzbromarone group (10 mg/kg positive control)	Yang et al. (2021)
*Polygonum cuspidatum* Siebold and Zucc. [Polygonaceae; *Polygoni cuspidate* rhizome et radix] extract	1.87, 3.73, 7.46 g/kg 8 w	Uric acid renal injury in rats (*in vivo*)	Upregulated the expression of AMPK, Inhibited the biological activity of TLR4, NLRP3, and MCP-1	Benzbromarone group (23.33 mg/kg positive control)	Ma et al. (2019)
*Morus alba* L. [Moraceae; Mori ramulus] extract	10, 20, 40 mg/kg 7 d	HUA mice (*in vivo*)	Upregulation adjustment of OAT1, downregulation adjustment of URAT1, GLUT9, upregulated expression of mOCT1/2 and mOCTN1/2	Probenecid group (100 mg/kg positive control)	Shi et al. (2012)
*Salvia plebeia* R.Br. [Lamiaceae; *Salviae* herba] extract	50, 100, 200 mg/kg 7 d	HUA mice (*in vivo*)	Inhibition of XOD activity	Allopurinol group (10 mg/kg positive control)	Kim et al. (2017)
*Smilax china* L. [Smilacaceae; *Smilacis chinae* rhizoma] total saponins	500 mg/kg 7 d	HUA mice (*in vivo*)	Downregulation of URAT1 and GLUT9, upregulation adjustment of OAT1 Inhibition of XOD activity	Allopurinol group (5 mg/kg positive control)	Wu et al. (2015)
*Leonurus japonicus* Houtt. [Lamiaceae; *Leonuri* herba] extract	50, 100, 200 mg/kg 7 d	HUA mice (*in vivo*)	Downregulation of URAT1 and GLUT9, upregulation adjustment of OAT1 and OCTN	Allopurinol group (5 mg/kg positive control)	Yan et al. (2014)
*Eucommia ulmoides* Oliv. [Eucommiaceae; Eucommiae cortex] extract	80, 160, 320 mg/kg in mice and 100, 200, 400 mg/kg in rats 7 d	HUA mice/rat (*in vivo*)	Downregulation of URAT1 and GLUT9, upregulation adjustment of OAT1 and OAT3	Allopurinol group (10 mg/kg positive control)	Fang et al. (2019)
*Ganoderma applanatum* extract	Water extract 175, 350, 700 mg/kg, ethanol extract 115, 230 and 460 mg/kg 1 w	HUA mice (*in vivo*)	Downregulation of URAT1 and GLUT9, upregulation adjustment of OAT1	Allopurinol group (5 mg/k) and Benzbromarone group (7.8 mg/kg positive control)	Yong et al. (2018b)
*Persicaria capitata* (Buch. -Ham. ex D.Don) H.Gross [Polygonaceae; *Persicaria capitata* herba] extract	Water extract 175, 350 and 700 mg/kg, ethanol extract 115, 230 and 460 mg/kg 7 d	HUA mice (*in vivo*)	Inhibition of XOD activity, downregulation of URAT1 and GLUT9	Allopurinol group (5 mg/kg) and Benzbromarone group (7.8 mg/kg positive control)	Zhang et al. (2021a)
*Rhus chinensis* Mill. [Anacardiaceae] extract	400 and 800 mg/kg 7 d	HUA mice (*in vivo*)	Inhibition of XOD activity, upregulation adjustment of ABCG2, downregulation of URAT1 and SLC2A9, elevated the levels of endogenous antioxidant enzymes, reducing the level of malondialdehyde, downregulation of NLRP3, ACS, and Caspase-3 and the levels of IL-1β and IL-6	Allopurinol group (5 mg/kg positive control)	Ma et al. (2024)
*Aster tataricus* L.f. [Asteraceae; *Asteris* radix et rhizoma] extract	100, 300, and 500 mg/kg 7 d	HUA rat (*in vivo*)	Inhibition of XOD activity, downregulation of URAT1 and GLUT9, upregulation adjustment of OAT1 and ABCG2, inhibition of TLRs/MyD88/NF-κB inflammatory signaling pathway	Allopurinol group (50 mg/kg positive control)	Jeong et al. (2022)
*Eruca sativa* Mill. [Brassicaceae] extract	10, 40, and 125 mg/kg 7 d	HUA rat (*in vivo*)	Treatment of hyperuricemia through hypoglycemic exertion	Allopurinol group (10 mg/kg, benzbromarone 10 mg/kg and probenecid 50 mg/kg positive control)	Teixeira et al. (2022)
*Cornus officinalis* Sieb. Et Zucc. [Cornaceae; *Corni* fructus] extract	250, 500, and 1,000 mg/kg 7 d	HUA mice (*in vivo*)	Inhibition of XOD activity	Allopurinol group (1 mg/kg positive control)	Wang et al. (2022b)
*Corylopsis coreana* Uyeki [Hamamelidaceae] extract	50 and 250 mg/kg 7 days	HUA mice (*in vivo*)	Inhibition of XOD activity	Allopurinol group (10 mg/kg positive control)	Yoon et al. (2016)
*Limonia acidissima* L. [Rutaceae; *Lycii* fructus] extract	100, 200, and 400 mg/kg 3 d	HUA rat (*in vivo*)	Downregulation of URAT1, elevated the levels of endogenous antioxidant enzymes	Allopurinol group (10 mg/kg positive control)	Yusnaini et al. (2023)
*Stevia rebaudiana* (Bertoni) Bertoni [Asteraceae; *Steviol*] extract	75, 150, and 300 mg/kg 3 d	HUA mice (*in vivo*)	Inhibition of XOD activity, upregulation adjustment of ABCG2 and OAT1, downregulation of URAT1 and GLUT9, elevated the levels of endogenous antioxidant enzymes, suppression of the inflammatory response	Allopurinol group (10 mg/kg positive control)	Mehmood et al. (2020)

**TABLE 2:** Mechanism of action of botanical drugs active metabolites in preventing HUA.

Bioactive metabolites	Dosage	Object	Effect/Mechanism	Controls	References
Rhubarbic Acid	75, 150, 300 mg/kg 2 w	HUA mice (*in vivo*)	Inhibition of XOD activity	Allopurinol group (10 mg/kg positive control)	Meng et al. (2015)
Emodin	10, 30, 50 mg/kg 3 d	HUA rat (*in vivo*)	Decreased the serum uric acid levels	Allopurinol group (7 mg/kg positive control)	Hou et al. (2023)
Berberine	50 mg/kg 1 w	HUA mice (*in vivo*)	Inhibition of XOD activity, downregulation of URAT1 and GLUT9	Febuxostat group (5 mg/kg positive control)	Xu et al. (2021)
Astilbin	1.25, 2.5, 5.0 mg/kg 4 w 5, 10, 20 mg/kg	HUA rat (*in vivo*) HUA mice (*in vivo*)	Decreased the serum uric acid	Allopurinol group (4 mg/kg positive control)	Chen et al. (2011a); Wang et al. (2016)
Apigenin	25, 50, 100 mg/kg 7 d	HUA mice (*in vivo*)	Improve UA metabolism, downregulation of URAT1 and GLUT9, upregulation adjustment of OAT1, inhibition of JAK2/STAT3 signaling pathway	Allopurinol group (5 mg/kg positive control)	Liu et al. (2022a)
Nuciferine	10, 20, 40 mg/kg 7 d	HUA mice (*in vivo*)	Downregulation of URAT1 and GLUT9, upregulation adjustment of OAT1	Allopurinol group (5 mg/kg positive control)	Wang et al. (2015)
Curcumin	20, 40 mg/kg 14 d	HUA mice (*in vivo*)	Inhibited serum and liver xanthine oxidase, inhibited the activation of NLRP3 inflammasome signaling	Allopurinol group (5 mg/kg positive control)	Chen et al. (2019)
Cordycepin	15, 30, 60 mg/kg 7 d	HUA mice (*in vivo*)	Decreased the URATl mRNA and protein, elevated the GLUT9 and OAT1 protein expressions	Allopurinol group (5 mg/kg) and Benzbromarone group (7.8 mg/kg positive control)	Yong et al. (2018a)
Epigallocatechin-3-gallate	10, 20, 50 mg/kg 7 d	HUA mice (*in vivo*)	Downregulation of URAT1 and GLUT9	Allopurinol group (5 mg/kg positive control)	Zhu et al. (2018)
Pallidifloside D	5, 10, 20 mg/kg 7 d	HUA mice (*in vivo*)	Decreased the URATl mRNA and mGLUT9	Allopurinol group (10 mg/kg positive control)	Wu et al. (2014)
Resveratrol	600 mg/kg 8 w	HUA mice (*in vivo*)	Enhanced intestinal UA degradation and reduced fecal UA concentration, increased abundance of Lactobacillaceae, Lactobacillales and *Lactobacillus*_sp	NA	Zhou et al. (2024)
Luteolin, Luteolin-4′-O-glucoside	20, 40, 100 mg/kg 7 d	HUA mice (*in vivo*)	Inhibition of XOD activity, downregulate the expression level of mURAT1	Allopurinol group (40 mg/kg positive control)	Lin et al. (2018)
Baicalein	100, 200 mg/kg 14 d	HUA mice (*in vivo*)	Downregulated renal GLUT9 and URAT1 mRNA and protein expression levels, inhibition of XOD activity	Allopurinol group (10 mg/kg positive control)	Chen et al. (2021)

**TABLE 3:** Mechanism of Traditional Chinese medicine formulas in preventing HUA.

Name of Formula	Prescription composition	Dosage	Object	Underlying mechanism	Controls	References
Ermiao Pill	*Phellodendron chinense* C.K.Schneid. [Rutaceae; *Phellodendri chinense* cortex] and *Atractylodes lancea* (Thunb.) DC. [Asteraceae; *Atractylodis* rhizoma]	1.10 g/kg 30 d	HUA rat (*in vivo*)	Regulation of phenylalanine metabolism, glycerophospholipid metabolism, tryptophan metabolism, purine metabolism, linoleic acid metabolism, lipid metabolism, taurine metabolism	NA	Shan et al. (2021)
						Gu et al. (2023)
Sanmiao Pill	*Phellodendron chinense* C.K.Schneid. [Rutaceae; *Phellodendri chinense* cortex], *Atractylodes lancea* (Thunb.) DC. [Asteraceae; *Atractylodis* rhizome] and *Coix lacryma-jobi* L. [Poaceae; Coicis semen]	4.68 g/kg 30 d	HUA rat (*in vivo*)	Regulation of amino acid metabolism, purine metabolism and energy metabolism	NA	Jiang et al. (2017)
Simiao Pill	*Phellodendron chinense* C.K.Schneid. [Rutaceae; *Phellodendri chinense* cortex], *Atractylodes lancea* (Thunb.) DC. [Asteraceae; *Atractylodis* rhizome], *Coix lacryma-jobi* L. [Poaceae; Coicis semen] and *Achyranthes bidentata* Blume [Amaranthaceae; *Achyranthis bidentatae* radix]	4.55, 9.10 and 18.2 g/kg 7 d	HUA mice (*in vivo*)	Inhibit the NLRP3 inflammasome and JAK2/STAT3 signaling, downregulation of URAT1 and GLUT9, upregulation adjustment of OAT	Allopurinol group (5 mg/kg positive control)	Zhang et al. (2023)
Er Ding Granules	*Viola yedoensis* Makino, Bot. Mag. [Violaceae; *Violae* herba], *Taraxacum mongolicum* Hand. [Asteraceae, *Taraxaci* herba], *Lobelia chinensis* Lour. [Campanulaceae; *Lobeliae chinensis* herba] and *Isatis indigotica* Fortune [Brassicaceae; *Isatidis* radix]	24 g/kg 5 d	HUA mice/rat (*in vivo*)	Inhibiting the expression of URAT1 mRNA and enhancing the expression of OAT3 mRNA	Benzbromarone group (20 mg/kg positive control)	Zuo et al. (2018)
Qu Zhuo Tong Bi Tang	*Smilax china* L. [Smilacaceae; *Smilacis chinae* rhizoma], *Dioscorea spongiosa* J. Q. Xi, M. Mizuno et W. L. Zhao [Dioscoreaceae; *Dioscoreae spongiosae* rhizoma], *Coix lacryma-jobi* L. [Poaceae; Coicis semen], *Curcuma longa* L. [Zingiberaceae; *Curcumae longae* rhizoma], *Corydalis yanhusuo* (Y.H.Chou and Chun C.Hsu) W.T.Wang ex Z.Y.Su and C.Y.Wu [Papaveraceae; *Cortdalis* rhizoma], *Verbesina siegesbeckia* Michx. [Asteraceae; *Siegesbeckiae* herba], etc.	25 g/kg 5 w; 18 g/kg 8 w	HUA rat (*in vivo*)	Regulation of amino acid metabolism, purine metabolism and energy metabolism, repair gut barrierregulate the differentiation of intestinal and splenic CD4^+^ T cells	Allopurinol group (10 mg/kg positive control); Benzbromarone group (6.5 mg/kg positive control)	Chen et al. (2016); Song et al. (2023)
Fangji Huangqi Tang	*Stephania tetrandra* S.Moore [Menispermaceae; *Stephaniae tetrandrae* radix], *Astragalus membranaceus* Bunge [Leguminosae; *Astragali* radix], *Glycyrrhiza uralensis* Fisch. ex-DC. [Fabaceae; *Glycyrrhizae* radix et rhizoma] and *Atractylodes macrocephala* Koidz. [Asteraceae; *Atractylodeis macrocephalae* rhizoma]	10,920, 5,460, 2,730 mg/kg 7 d	HUA mice (*in vivo*)	Upregulation adjustment of OAT1, OAT3, ABCG2	Allopurinol group (5 mg/kg positive control)	Xing et al. (2020)
Shi Wei Ru Xiang powder	*Phyllanthus emblica* L. [Phyllanthaceae; Thylianthi fructus], *Cassia obtusifolia* L. [Caesalpiniaceae; Cassiae semen], *Boswellia carterii Birdw., Tinospora sinensis* (Lour.) Merr. [Menispermaceae; Tinosporae caulis], *Terminalia chebular* Retz., [Combretaceae; Chebulae fructus], *Aucklandia lappa* Decne. [Asteraceae; Aucklandiae radix], *Terminalia billirica* (Gaert.) Roxb., *Abelmoschusmanihot* L.) Medic., *Adhatoda vasica Nees.* And *Shilajit*	390, 780, 1,560 mg/kg 28 d	HUA mice (*in vivo*)	Regulation of the MAPK signaling pathway, NF-κB signaling pathway and NOD-like receptor signaling pathway	Allopurinol group (5 mg/kg positive control)	Li et al. (2022)
Cheqianzi Tang	*Plantago asiatica* L. [Plantaginaceae; Plantaginis Semen], *Achyranthes bidentata* Blume [Amaranthaceae; *Achyranthis bidentatae* radix], *Typha angustifolia* L. [Typhaceae; Typhae pollen] and *Morus alba* L. [Moraceae; Mori cortex]	4.32 g/kg 7 d	HUA rat (*in vivo*)	Increasing ABCG2 expression at both the mRNA and protein levels, downregulating the mRNA and protein expression of NLRP3 expression	Allopurinol group (20 mg/kg positive control)	Meng et al. (2023)
Zexie Tufuling Tang	*Alisma orientale* (Sam.) Juz. [Alismataceae; *Alismatis* rhizome], and *Smilax glabra* Roxb. [Smilacaceae; *Smilacis glabrae* rhizoma]	First stage Alismatis rhizome (0.50, 2.00 g/kg); *Smilacis glabrae* rhizoma (1.25, 5.00 g/kg) Second stage *Alismatis* rhizome + *Smilacis glabrae* rhizoma (1.00 + 1.00, 2.00 + 1.00, 1.00 + 1.00 and 2.00 + 2.00 g/kg) 45 d	HUA rat (*in vivo*)	Inhibiting activity of XOD, down-regulating the expression of URAT1	Allopurinol group (2.7 mg/kg positive control)	Cheng et al. (2019)

### 3.1 Single botanical drug extracts

#### 3.1.1 *Astragalus membranaceus* Bunge [Fabaceae; *Astragali* radix]

*Astragalus membranaceus* Bunge is a medicinal plant rich in flavonoids, saponins, polysaccharides, and triterpenoids, and exhibits diverse biological activities. Modern pharmacological studies have demonstrated its anti-inflammatory, antioxidant, and immunomodulatory effects (Durazzo et al., 2021). Moreover, astragalus exerts renoprotective effects and regulates intestinal flora (Shahzad et al., 2016). In a yeast- and potassium oxybate-induced hyperuricemic mouse model (HUA mice), fermented astragalus (BFA) effectively reduced blood urea nitrogen (BUN) and serum creatinine (SCr) levels while improving renal morphology. BFA upregulated renal ABCG2 protein expression to enhance uric acid excretion and downregulated renal URAT1 and GLUT9 expression to inhibit uric acid reabsorption. Additionally, BFA attenuated the renal inflammatory response by preserving the integrity of the intestinal mucosal barrier and reducing lipopolysaccharide-binding protein production. Metabolomic analysis revealed that BFA ameliorated HUA by suppressing boswellic acid production while enhancing the abundance of beneficial monocytic butyric acid bacteria, visceral odoriferous bacteria, Tanaka bacteria as well as fatty acid biosynthesis to maintain urea cycle homeostasis (Wang et al., 2023).

#### 3.1.2 *Atractylodes macrocephala* Koidz. [Asteraceae; *Atractylodis* rhizome]

Dried rhizomes of Atractylodes macrocephala Koidz. is commonly employed in traditional medicine for the treatment of gout due to its diuretic and anti-dampness properties (Zhang et al., 2021). In a HUA rat model established by combining otacid potassium with yeast powder solubilization, it was observed that C. diff effectively reduced serum levels of uric acid (UA), adenosine deaminase (ADA), and XOD in hypouricemic rats. Hematoxylin-eosin staining revealed that Atractylodis macrocephalae rhizome improved renal tubular dilatation and interstitial inflammatory cell infiltration in hypouricemic rats. Furthermore, Atractylodis macrocephalae rhizome exhibited anti-inflammatory activity by downregulating macrophage interleukin-1β (IL-1β) and tumor necrosis factor-α (TNF-α) levels, activating the Adenosine 5′-monophosphate-activated protein kinase/Silent mating type information regulation 2 homolog-1 (AMPK/SIRT1) signaling pathway, inhibiting nuclear factor kappa-B (NF-κB) activation, and suppressing macrophage polarization towards a pro-inflammatory phenotype (Qian et al., 2023).

#### 3.1.3 *Cichorium glandulosum* Boiss. & A. Huet [Asteraceae; *Cichorii herba cichorii* radix]

Cichorii herba cichorii radix is the dried above-ground part or root of Cichorium glandulosum Boiss. et Huet or Cichorrium intybus, belonging to the Asteraceae family. Recent pharmacological studies have demonstrated Cichorii herba cichorii radix’s potent anti-inflammatory, antioxidant, hypoglycemic, hypolipidemic, and intestinal flora regulating effects (Azay-Milhau et al., 2013; Chen et al., 2017; Boghrati et al., 2021). Bian M et al. discovered that chicory effectively reduces serum uric acid levels by inhibiting xanthine oxidase activity and upregulating expression of the uric acid transporter ABCG2 in the intestine (Bian et al., 2018). To further investigate the potential relationship between chicory treatment and regulation of intestinal flora in HUA, Bian M induced HUA in quails using a high purine diet based on previous research findings. The study revealed that chicory intervention significantly decreased serum uric acid levels while increasing fecal uric acid levels, promoting repair of intestinal mucosal damage and improving intestinal barrier permeability. Moreover, analysis through 16S rRNA sequencing indicated that chicory restored gut microbiota balance by enhancing probiotic flora (Bifidobacterium, *Salmonella* family) and reducing pathogenic flora (Pylori family). This restoration was accompanied by downregulation of serum lipopolysaccharide (LPS) levels as well as renal Toll-like receptor 4/nuclear factor-kappa B inflammatory pathways, ultimately facilitating renal excretion of uric acid via attenuation of the LPS/Toll-like receptor 4 axis-mediated inflammatory response (Bian et al., 2020).

#### 3.1.4 *Plantago asiatica* L. [Plantaginaceae; Plantaginis Semen]

Plantaginis Semen is the dried mature seeds of Plantago asiatica L. or Plantago depressa Willd. a herb commonly employed for uric acid reduction (Zeng et al., 2018; Sun et al., 2019). The phosphoinositide 3-kinase/Protein kinase B (PI3K/Akt) signaling pathway, an inflammatory pathway implicated in severe kidney injury and indirect modulation of uric acid excretion, can be inhibited by Plantago to ameliorate nephropathy and enhance uric acid excretion (Dragos et al., 2020; Santos et al., 2020; Zhao et al., 2020). Furthermore, hyperuricemia has been associated with lipid metabolism (Guo et al., 2020), but it remains unknown whether lipid metabolism plays a role in the therapeutic effects of Plantago on hyperuricemia treatment. Previous studies have shown that Plantago extract improves lipid accumulation in high-fat diet-induced obese mice (Yang et al., 2017), while another study demonstrated that Plantago accelerates lipolysis through regulation of lipid metabolism disorders in a mouse model of hyperuricemia induced by potassium oxybate gavage; additionally, anti-hyperuricemic effects were observed through downregulation of URAT1 expression and inhibition of the PI3K/Akt inflammatory pathway (Yang et al., 2021).

#### 3.1.5 *Polygonum cuspidatum* Siebold & Zucc. [Polygonaceae; *Polygoni cuspidate* rhizome et radix]

The Polygoni cuspidate rhizome et radix is derived from the dried rhizomes and roots of Polygonum cuspidatum Sieb.et Zucc, a perennial herb of the Polygonicuspidate family. PC has anti-inflammatory, antibacterial, antiviral, antioxidant, hypoglycemic and anti-hyperuric acid pharmacological activities (Ammar et al., 2022; Bian et al., 2022; Lin et al., 2022). Metabolomics is an emerging discipline in the 20th century and is of great importance in elucidating the mechanisms of pharmacological effects of Chinese medicines (Wu et al., 2019). Recent reports have found that polydatin can improve and treat HUA through amino acid metabolism, lipid metabolism and energy metabolism (Ge et al., 2023). Ma et al. found that PC could upregulate the expression of Adenosine 5′-monophosphate-activated protein kinase (AMPK) and its downstream molecule FOXO3α, and inhibit the bioactivity of Toll-likereceptor4 (TLR4), NOD-like receptor thermal protein domain associated protein 3 (NLRP3) and Monocyte Chemoattractant Protein-1 (MCP-1), key signaling molecules of the immune inflammatory network pathway to improve HUA-mediated immunoinflammatory metabolic kidney damage (Liu et al., 2005; Ma et al., 2019). Hu et al. isolated two active compounds from PC: 1-(4-hydroxy-2-methoxyphenyl)-2-(4-hydroxy-3,5-dimethylphenyl) butane-1,2,3-triol and 1-(4-hydroxy-2-methoxyphenyl)-2-(4-hydroxy-3,5-dimethylphenyl)-3-methylbutane-1,2-diol. *In vitro* and *in vivo* experiments have demonstrated the ability of both compounds to reduce UA levels and improve renal morphological and pathological changes through competitive inhibition of XOD activity. In addition, the mechanisms of anti-HUA are closely related to galactose metabolism, taurine and hypotaurine metabolism, purine metabolism and energy metabolism (Hu et al., 2023).

#### 3.1.6 *Smilax china* L. [Smilacaceae; *Smilacis chinae* rhizoma]

*Smilacis chinae* rhizoma, the dried rhizome of *Smilax china* L., a member of the lily family, possesses therapeutic properties including dampness relief, turbidity removal, wind and paralysis dispelling, as well as detoxification and blood stasis dispersion. It exhibits anti-inflammatory, antioxidant, anticancer, hypoglycemic, and diuretic activities (Wang et al., 2013; Hooda et al., 2014; Bao et al., 2018; Xu et al., 2022b). Chen investigated different fractions of sarsaparilla using petroleum ether, chloroform, ethyl acetate n-butanol and ethanol in HUA rats. The ethyl acetate fraction demonstrated significant uric acid-lowering effects. Caffeic acid, resveratrol rutin and oxidized resveratrol isolated from the ethyl acetate fraction exhibited *in vitro* inhibition of xanthine oxidase activity (Chen et al., 2011b). Wu previously reported that sarsaparilla saponin significantly reduced serum uric acid levels in a mouse model of HUA. Further investigation revealed that sarsaparilla saponin co-regulated renal URAT1 and GLUT9 expression in mice by upregulating OAT1 while inhibiting XOD (Wu et al., 2015). Hong et al. (2014) employed Liquid Chromatograph Mass Spectrometer analysis combined with bioinformatics to identify differential proteins in kidney tissue from sarsaparilla-intervened HUA rats. Sarsaparilla was found to upregulate catalase expression for alleviating oxidative stress levels.

#### 3.1.7 *Morus alba* L. [Moraceae; Mori ramulus]

Mori ramulus (MR) is a branch of the mulberry tree, extensively utilized in traditional medicine as an anti-rheumatic agent. MR contains various active constituents, including flavonoids, phenylpropanoids, and coumarins, exhibiting pharmacological activities such as antioxidative, anti-inflammatory, anti-hyperlipidemic, and anti-hyperglycemic effects (Xiang et al., 2021). Shi et al. (2012) discovered that the alcoholic extract derived from Morus alba exhibited significant efficacy in reducing serum uric acid levels and increasing 24-hour urine uric acid excretion as well as partial uric acid excretion in mice with HUA. This extract effectively downregulated renal mURAT1 and mGLUT9 expression, while up-regulating the expression of mOAT1, thereby enhancing uric acid excretion. Furthermore, EMR treatment resulted in reduced serum creatinine and BUN levels, increased creatinine clearance, upregulated the expression of mOCT1/2, and facilitated HUA improvement.

#### 3.1.8 *Salvia plebeia* R. Br. [Lamiaceae; *Salviae* herba]

*Salvia plebeia* R. Br. (SP) is a Labiatae herbaceous plant, widely distributed in China, Korea, Japan, India, Iran, and Australia. In China, it is commonly referred to as “lychee grass” and has been extensively used in traditional medicine for treating ascites swelling and nephritic edema. Numerous studies have documented the anti-inflammatory, antioxidant, antibacterial, and antiviral properties of SP (Chen and Kang, 2014; Jin et al., 2015). Recent research focusing on xanthine oxidase inhibitors has revealed that SP exhibits potent inhibitory effects on XOD activity. Kim et al. demonstrated that baicalin and lignans present in SP significantly contribute to the inhibition of XOD activity with IC50 values below 5 μm (Kim et al., 2017).

### 3.2 Single bioactive metabolites

#### 3.2.1 Rhubarbic acid/Emodin

*Rhei* radix et rhizome, a traditional Chinese medicine, is derived from the dried roots and rhizomes of *Polygonum palmatum*, *Rheum palmatum*, *Rheum tanguticum*, or *Rheum officinale* in the family *Polygonaceae*. *Rhubarb* contains anthraquinones such as rhubarbic acid, rhubarbol, and rhubarb phenol which possess antibacterial, anti-inflammatory, antiviral, antioxidant properties as well as exhibit effects against HUA and renal fibrosis (Mueller-Heupt et al., 2022). Meng et al. (2023) demonstrated that Rhubarbic Acid has notable uric acid-lowering and nephroprotective effects on HUA mice by significantly reducing serum uric acid levels along with serum creatinine and blood urea nitrogen levels while inhibiting XOD activity in mouse liver. Emodin is an anthraquinone from rhubarb that has anti-inflammatory, detoxifying, and gut motility-promoting effects; in addition, some studies have reported that emodin can also inhibit XOD activity (Shi et al., 2014). A recent study by Hou et al. (2023) found that rhodopsin can significantly reduce serum uric acid levels, promote uric acid excretion, and thus play a role in the treatment of hyperuricemia.

#### 3.2.2 Berberine

*Phellodendri chinense* cortex (PC), commonly known as “Chuan Huang Bai,” is extensively utilized for the treatment of damp-heat diarrhoea, jaundice and dysentery, pyorrhoea, and astringent pain. PC has been traditionally employed as a medicinal remedy for gout and hyperuricemia, such as in Ermiao Wan, Sanmiao Wan, and Ventilation Soup. Pharmacological investigations have revealed that PC contains diverse bioactive constituents including alkaloids and flavonoids. Notably, berberine, an alkaloid group present in PC, has recently demonstrated anti-hyperuricemic activity by modulating the expression of URAT1 and GLUT9 transporter proteins along with XOR activity regulation (Li et al., 2021; Naz et al., 2021). Xu et al. (2021) investigated the hypouricemic and nephroprotective effects of dihydroberberine in hyperuricemic mice and observed significant reduction in serum uric acid levels as well as XOD levels while inhibiting hepatic XOD activity and ADA activities. Furthermore, they found downregulation of renal XOD mRNA and protein expression.

#### 3.2.3 Astilbin

Extracted from sarsaparilla rhizomes, Astilbin is an active flavonoid compound that is widely used in traditional Chinese medicine therapy for its anti-arthritic, anti-hepatic, and anti-kidney injury effects. Wang et al. (2016), in order to study the effect of Astilbin on potassium oxybate-induced hyperuricemia mice and its mechanism of action. The results showed that Astilbin significantly reduced serum uric acid (Sur) levels, and its effects were associated with the inhibition of GLUT9 and URAT1, expression and the upregulation of ABCG2, OAT1/3 and OCT1 expression. In addition, Aastilbin inhibited the activation of Janus kinase 2/signal transducer and activator of transcription 3 (JAK2/STAT3) cascade and overexpression of suppressor of cytokine signaling 3 (SOCS3), and exerted nephroprotective effects by inhibiting oxidative stress.

#### 3.2.4 Apigenin

Apigenin (4,5,7 -trihydroxyflavone) is a naturally occurring flavonoid that is mainly derived from *Apium graveolens* L. (celery), but is also found in a wide variety of plants, fruits, and vegetables. There is growing evidence that apigenin attenuates UA in mice with chromosome- or potassium oxybate-induced HUA (Chesworth et al., 2021). Previously, it has been shown that apigenin can reduce serum uric acid levels, decrease the levels of GLUT9 and URAT1 transport proteins, increase the levels of OAT1 transport proteins, and inhibit the JAK2/STAS3 signaling pathway in a mouse model of acute hyperuricemia induced by potassium oxybate and Hypoxanthine, thus exerting a therapeutic effect on hyperuricemia (Liu et al., 2022).

### 3.3 Traditional Chinese medicine formulas

Throughout the millennia of Chinese culture, Chinese medicine has become an integral component of the comprehensive medical system (Xu et al., 2013). Chinese medicine formulas are not haphazardly concocted but have evolved over thousands of years through clinical practice. According to Chinese medicine, hyperuricemia primarily arises from dampness and heat, thus treatment predominantly focuses on clearing heat and alleviating dampness using formulations like Ermiao Wan, Er Ding Granules, and Qu Zhuo Tong Bi Tang. Our analysis of commonly employed traditional Chinese medicine prescriptions has revealed various approaches for managing hyperuricemia (Table 3).

#### 3.3.1 Pharmacological mechanism of Ermiao Pill and similar formulations in treating HUA

Ermiao Wan (EMW) is derived from Zhu Zhenheng’s “Danxi Xinfa” and is utilized for the treatment of damp-heat infiltration, damp-heat banding, and gonorrhea. The complete formula comprises two botanical drugs, namely, *Phellodendri chinense* cortex and Atractylois rhizoma. Subsequently, based on their remarkable efficacy in reducing uric acid levels, Sanmiao Wan (SMW) and Simiao Pill were developed by later medical practitioners to enhance therapeutic outcomes. Previous studies have demonstrated that *Phellodendri chinense* cortex, *Atractylois* rhizoma and Coicis semen possess diuretic properties with the ability to eliminate dampness. Additionally, hyssop has traditionally been recognized for its downward-moving effect and frequent usage as a menstruation-inducing herb (Zhao et al., 2014). Huang et al. (2019) conducted an investigation into the comprehensive composition and mechanism of action of EMW in treating HUA, identifying 24 alkaloids, 46 volatile components, 15 organic acids, 4 terpenoids, 3 lactones, 3 glycosides along with other compounds within this formulation. Metabolomic analysis revealed that EMW exerts anti-HUA effects through the regulation of multiple metabolic pathways including glycerolipid metabolism amino acid metabolism prochlorogenic bile acid metabolism taurine and hypotaurine metabolism as well as purine metabolism. Several studies have elucidated the mechanism of action of EMW in ameliorating HUA from a metabolomics perspective. Following EMW intervention, there was a significant reduction in serum uric acid levels. Metabolomics analysis identified 11 biomarkers exhibiting a reversal trend. Pathway analysis revealed that EMW may exert therapeutic effects on HUA rats through pathways such as phenylalanine metabolism, glycerophospholipid metabolism, tryptophan metabolism, and lipid metabolism (Shan et al., 2021; Gu et al., 2023). Hyperuricemia can contribute to the development of chronic kidney disease and cardiovascular disease. Guo et al. (2015) investigated the combined protective effects of Siwu Tang and EMW on HUA and renal injury and demonstrated that this combination significantly reduced serum levels of uric acid, creatinine, triglycerides, and urea nitrogen. The underlying mechanism may involve decreased renal xanthine oxidase activity and upregulation of OAT1 and OAT3 expression. Serum metabolomic analysis revealed that SMW could partially regulate purine metabolism, amino acid metabolism, and energy metabolism to reverse the pathological process associated with HUA (Jiang et al., 2017). Simiao Wan is utilized as a herbal formulation for adjunctive therapy in gout. A study conducted by Cao et al. (2021) demonstrated that Simiao Wan exhibits potential in ameliorating MSU-induced gouty arthritis and inhibiting hyperuricemia, possibly through the activation of the PI3K/Akt signaling pathway to promote M2 polarization. Zhang et al. (2023) discovered that Simiao powder could attenuate HUA in mice by reducing the expression of URAT1, GULT9, NLRP3, Phospho-Janus Kinase-2/Janus Kinase-2 (P-JAK2/JAK2), and Phospho-signal transducer and activator of transcription-3/signal transducer and activator of transcription-3 (P-STAT3/STAT3) in renal tissues; this mechanism of action may be associated with the suppression of NLRP3 inflammasome and Janus Kinase-2/signal transducer and activator of transcription-3 (JAK2/STAT3) signaling pathway. In summary, EMW and its analogs exhibit therapeutic potential against HUA through four pathways: inhibition of renal XOD activity; upregulation of transporter proteins OAT1 and OAT3; regulation of purine metabolism and amino acid metabolism; modulation of the PI3K/Akt and JAK2/STAT3 signaling pathways.

#### 3.3.2 Pharmacological mechanism of Er Ding granules in treating HUA

The composition of Er Ding Granules includes four botanical drugs, namely, *Violae* herba, *Taraxaci* herba, *Lobeliae chinensis* herba and *Isatidis* radix, all of which were initially documented in the Shen Nong Ben Cao Jing. It is well-documented that *Violae* herba, *Lobeliae chinensis* herba and *Isatidis* radix possess anti-inflammatory and antibacterial properties. Additionally, Dandelion exhibits diuretic effects by increasing renal urine excretion. Zuo et al. (2018) demonstrated that treatment with Er Ding Granules significantly reduced serum uric acid levels in mice while down-regulating URAT1 mRNA expression in the kidneys of hyperuricemic mice and enhancing OAT3 mRNA expression. Zhang et al. (2019) divided the complete formula into water extracts as well as 50% ethanol and 95% ethanol extracts to investigate their anti-hyperuricemic activity. The results revealed a significant dose-dependent effect on serum uric acid levels for the 50% ethanol extract. Mechanistic studies indicated that the anti-hyperuricemic effect of the 50% ethanol extract primarily involved downregulation of GLUT9 and URAT1 protein expression along with upregulation of OAT1 protein expression. Downregulation of reabsorption proteins GLUT9 and URAT1 along with upregulation of secretory proteins OAT1 and OAT3 represent key mechanisms underlying the therapeutic effects exerted by Er Ding Granules on hyperuricemia.

#### 3.3.3 Pharmacological mechanism of Qu Zhuo Tong Bi Tang in treating HUA

The empirical formula Qu Zhuo Tong Bi Tang (QZTBD) demonstrates definite clinical efficacy in the treatment of HUA and gout, as it effectively enhances kidney function, improves blood circulation, and alleviates pain. This formula comprises *Smilacis chinae* rhizoma, *Dioscoreae spongiosae* rhizoma, Coicis semen, *Curcumae longae* rhizoma, *Cortdalis* rhizoma and Siegesbeckiae herba (Wen et al., 2021). Chen et al. (2016) employed serum metabolomics to investigate the impact of QZTBD on serum urine and other metabolites in HUA-induced rats. Their findings confirm that QZTBD significantly reduces serum uric acid levels by regulating amino acid metabolism, purine metabolism, and energy metabolism mechanisms. Song et al. utilized a combined analysis of network pharmacology and intestinal flora to elucidate the mechanism of action underlying QZTBD’s therapeutic effects against HUA. The results demonstrate that QZTBD has the potential to enhance the abundance of Allobaculum and Candidatus sacchairmonas while rectifying abnormal amino acid patterns. Additionally, it repairs compromised intestinal barriers by restoring Th17/Treg cell balance through modulation of the PI3K-AKT-mTOR pathway; furthermore reducing inflammatory factors such as IL-1β, interleukin-6 (IL-6), TNF-α, and interleukin-17 (IL-17) levels (S et al., 2023). In summary, QZTBD exerts its therapeutic effects on HUA through regulation of amino acid metabolism, purine metabolism, and energy metabolism. Additionally, it restores intestinal barrier integrity, inhibits the PI3K-AKT signaling pathway.

#### 3.3.4 Pharmacological mechanism of Fangji Huangqi Tang in treating of HUA

The prescription Fangji Huangqi Tang is derived from the synopsis of the Golden Chamber written by Zhang Zhongjing, and it primarily focuses on treating Feng Shui or rheumatic diseases. Composed of *Stephaniae tetrandrae* radix, *Astragali* radix, *Glycyrrhizae* radix et rhizoma and *Atractylodeis macrocephalae* rhizoma, this representative prescription aims to replenish qi and promote water balance. Previous studies have reported that *Stephaniae tetrandrae* radix and *Astragali* radix possess diuretic effects which can improve kidney function. Additionally, *Atractylodeis macrocephalae* rhizoma and *Glycyrrhizae* radix et rhizoma have been found to reduce serum uric acid levels while providing renal protection (Aksoy et al., 2012; Wang et al., 2018; Wei et al., 2018). Recent research has demonstrated that Fangji Huangqi Tang significantly lowers serum uric acid and creatinine levels while inhibiting IL-1β in both serum and kidneys of Hua mice. Furthermore, it effectively reduces NF-kB protein expression. Analysis of renal uric acid-related transporters revealed that Fangji Huangqi Tang upregulates the protein expression levels of renal OAT1, OAT3, and ABCG2 (Xing et al., 2020). When used individually as single botanical drug or combined together in Fangji Huangqi Tang formulae, *Stephaniae tetrandrae* radix, *Astragali* radix and *Atractylodeis macrocephalae* rhizoma, exhibit diuretic effects with enhanced efficacy when combined together (Lin et al., 2015). Moreover, their combination significantly upregulates the expression of renal uric acid secretion proteins OAT1, OAT3, and ABCG2 while substantially reducing organismal uric acid levels.

#### 3.3.5 Pharmacological mechanism of Shi Wei Ru Xiang powder in the treating of HUA

Shiwei Ruxiang powder, a traditional Tibetan medicine, is commonly utilized in traditional Chinese medicine for the treatment of HUA. In a study conducted by Li et al. (2022), network pharmacology and experimental validation were employed to investigate the effects of SWS on HUA in mice. The findings demonstrated that SWS ameliorated HUA through modulation of pivotal signaling pathways encompassing MAPK, nuclear factor κB (NF-κB), and NOD-like receptor signaling pathways.

#### 3.3.6 Pharmacological mechanism of Cheqianzi Tang in the treating of HUA

In the Song Dynasty’s “Shengji Zonglu,” the Cheqianzi Tang (CQD) was discovered to possess the following therapeutic properties: heat-clearing and detoxification, dampness-promoting and dehumidifying effects. It has demonstrated a positive curative impact on hyperuricemia. The composition of CQD comprises Plantaginis semen, *Achyranthis bidentatae* radix, Typhae pollen, and Mori cortex. Studies have reported that psyllium decoction can ameliorate hyperuricemia by activating ABCG2, which facilitates uric acid excretion, as well as inducing downregulation of inflammatory and apoptotic factors mediated by inflammasome NLRP3 (J et al., 2023).

## 4 Discussion

This article provides a comprehensive review of the botanical drugs commonly used for treating HUA in rat and mice models and presents research findings that demonstrate the efficacy of everal specific botanicals, bio-metabolites and herbal formulations in inhibiting hepatic XOD activity, modulating renal uric acid transport proteins, suppressing inflammatory response pathways, and regulating product metabolism.

**FIGURE 3:**
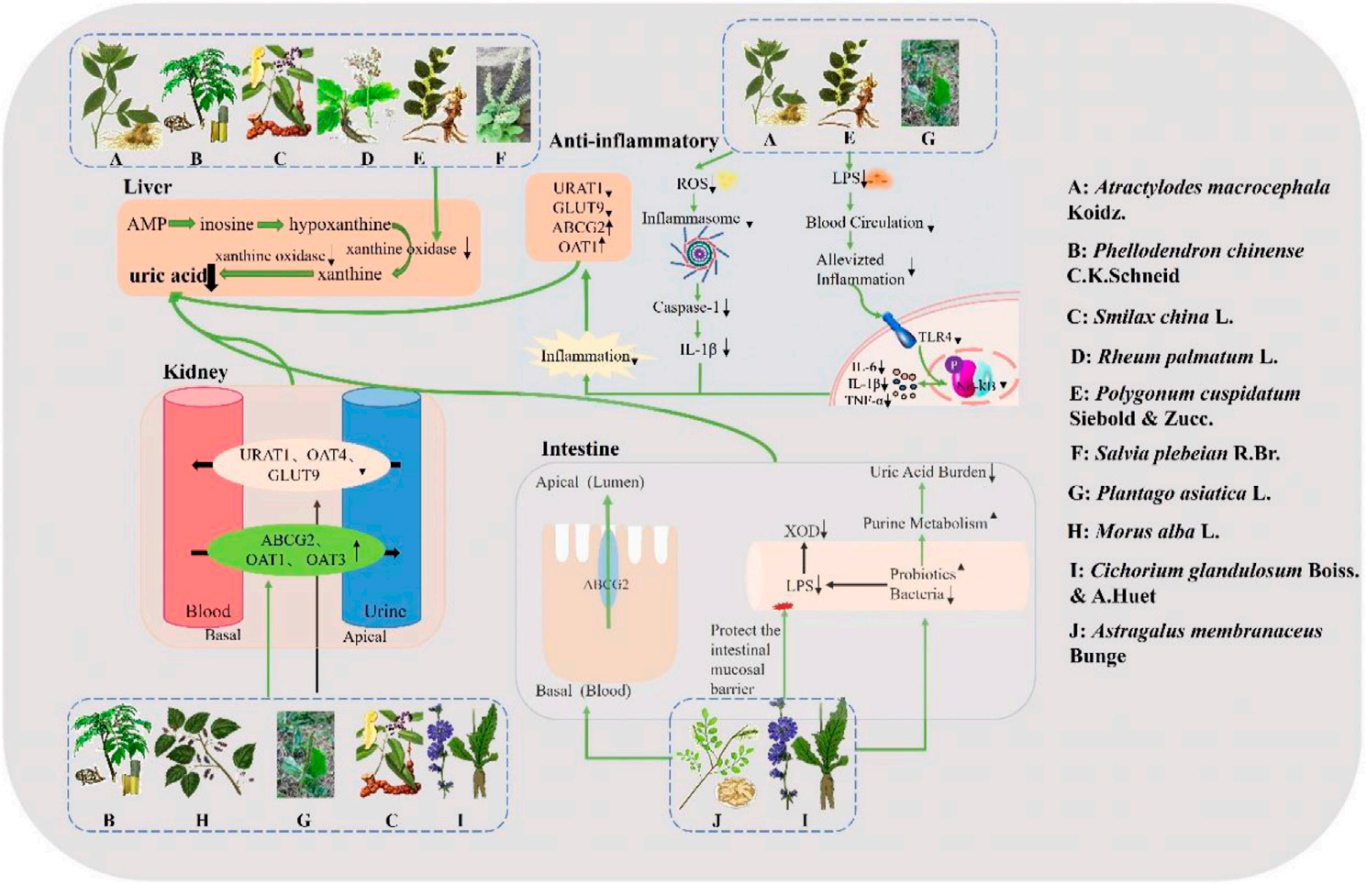
Mechanism of action of traditional Chinese medicine in the treatment of HUA. *Atractylodes macrocephala* Koidz., *Phellodendron chinense* C. K. Schneid., *Smilax china* L., *Rheum palmatum* L., *Polygonum cuspidatum* Siebold & Zucc., and *Salvia plebeia* R. Br. exhibit direct inhibitory effects on XOD activity for uric acid reduction. *Atractylodes macrocephala* Koidz., *Polygonum cuspidatum* Siebold & Zucc. and *Plantago asiatica* L. exert their hypouricemic effects by suppressing the TLR4/NF-kB signaling pathway and NLRP3 inflammasomes. *Phellodendron chinense* C.K.Schneid., *Morus alba* L., *Plantago asiatica* L., *Smilax china* L. and *Cichorium glandulosum* Boiss. & A. Huet reduce uric acid levels by downregulating reabsorption-associated proteins and upregulating excretion-associated proteins in the kidney. *Astragalus membranaceus* Bunge and *Cichorium glandulosum* Boiss. & A. Huet directly target uric acid excretory proteins in the intestine or demonstrate hypouricemic effects through regulation of intestinal flora and protection of the intestinal mucosal barrier.

Xanthine oxidase, an enzyme crucial in the pathogenesis of HUA and uric acid homeostasis, catalyzes the oxidation of hypoxanthine to xanthine and further to uric acid (Sang et al., 2017). Among the aforementioned botanical drugs, *Atractylodes macrocephala* Koidz., *Phellodendron chinense* C. K. Schneid.*, Polygonum cuspidatum* Siebold & Zucc., *Smilax china* L., *Rheum palmatum* L. and *Salvia plebeia* R. Br. exhibit inhibitory effects on XOD activity. Renal transporter proteins associated with uric acid metabolism can be categorized into two groups: URAT1, OAT4, GLUT9 as urate reabsorption transporters; OAT1, OAT3, MRP4/ABCC4, and ABCG2 as urate excretion transporters. Altered expression and function of these transporter proteins are implicated in HUA development (Zhao et al., 2022). In addition to *Phellodendron chinense* C.K.Schneid and *Cichorium glandulosum* Boiss. & A. Huet which inhibit hepatic XOD activity while modulating renal uric acid-related transporter proteins; *Plantago asiatica* L., *Astragalus membranaceus* Bunge and *Morus alba* L. also regulate serum uric acid levels by modulating renal uric acid-related transporter proteins. Severe HUA can lead to renal inflammation and gut microbial dysbiosis; hence targeting inflammatory response inhibition along with modulation of gut microbial populations may offer a novel approach for improving HUA management (Zhao et al., 2022). Among the ten botanical drugs mentioned above, *Astragalus membranaceus* Bunge and *Cichorium glandulosum* Boiss. & A. Huet can enhance hyperuricemia (HUA) by augmenting probiotics such as Bifidobacterium and *Bacillus* tansy, thereby fortifying the intestinal mucosal barrier. Additionally, *Cichorium glandulosum* Boiss. & A. Huet exhibits inhibitory effects on the TLR4/NF-kB inflammatory signaling pathway to impede the progression of HUA-induced nephritis. Conversely, *Atractylodes macrocephala* Koidz., *Plantago asiatica* L. and *Polygonum cuspidatum* Siebold & Zucc. mitigate further advancement of HUA primarily through suppression of NF-kB and PI3K/Akt inflammatory signaling pathways along with NLRP3 inflammatory vesicles. In summary, Chinese medicine effectively addresses HUA via four principal mechanisms of action (Figure 3).

Traditional Chinese medicine has multiple advantages in the treatment of hyperuricemia. Firstly, it employs a multi-pathway, multi-target treatment strategy, which effectively reduces uric acid levels and improves overall health. Chinese herbal medicine achieves therapeutic goals through various pathways such as regulating uric acid production, increasing uric acid excretion, and reducing uric acid absorption. This comprehensive action can provide more lasting and balanced therapeutic effects.

Secondly, Chinese herbal medicine is mostly derived from natural sources and has undergone extensive historical validation and clinical practice. These medicines often have fewer side effects because they are more compatible with the human body’s natural processes. In contrast, many conventional uric acid-lowering drugs may cause common side effects such as indigestion and headaches (Wang et al., 2020).

In addition, Chinese herbal medicine is often used in complex formulations, which not only enhances efficacy but also reduces the potential toxicity of individual components. For example, Ge Gen Qin Lian Tang, a classic Chinese herbal formula, is widely used for treating hyperuricemia. It has been proven effective in lowering uric acid levels and is considered safe (Wang et al., 2023).

This article summaries research on the potential and mechanisms of herbal medicine for the treatment of hyperuricemia. The studies focused on reducing XOD activity, regulating uric acid transporter proteins, influencing inflammatory signaling pathways, and regulating gut microbial homeostasis. Although the studies on regulating intestinal microbial balance and upstream regulators of uric acid transporter proteins still need to be deepened, traditional Chinese medicines show good prospects for application in rat and mouse models. Overall, the treatment of hyperuricemia with traditional Chinese medicines not only demonstrates significant advantages in reducing uric acid levels, but also improves overall health and reduces the risk of complications. Pharmacological studies, clinical evidence and historical applications support the safety and efficacy of TCM in the treatment of hyperuricemia.

## Figures and Tables

**Table:** 

**HUA**	Hyperuricemia
**TCM**	Traditional Chinese herbal medicine
**XOD**	Xanthine oxidase
**URAT1**	Urate anion transport protein 1
**OAT4**	Organic anion transport protein 4
**GLUT9**	Glucose transport protein 9
**OAT1**	Organic anion transporters 1
**OAT3**	Organic anion transporters 3
**MRP4/ABCC4**	Multidrug resistance protein 4
**BCRP/ABCG2**	Breast cancer resistance protein
**BFA**	Fermented astragalus
**BUN**	Blood urea nitrogen
**SCr**	Serum creatinine
**UA**	Uric acid
**ADA**	Adenosine deaminase
**IL-1β**	Interleukin-1β
**TNF-α**	Tumor necrosis factor-α
**NF-κB**	Nuclear factor kappa-B
**LPS**	Lipopolysaccharide
**PC**	*Phellodendri Cortex*
**AMPK**	Adenosine 5′-monophosphate-activated protein kinase
**TLR4**	Toll-likereceptor4
**NLRP3**	NOD-like receptor thermal protein domain associated protein 3
**MCP-1**	Monocyte Chemoattractant Protein-1
**SP**	*Salvia plebeia* R. Br
**EMW**	Ermiao Wan
**SMW**	Sanmiao Wan
**QZTBD**	Qu Zhuo Tong Bi Tang
**IL-6**	Interleukin-6
**IL-17**	Interleukin-17
**CQD**	Cheqianzi Tang
**SIRT1**	Silent mating type information regulation 2 homolog-1
**PI3K**	Phosphoinositide 3-kinase
**Akt**	Protein kinase B
**P-JAK2**	Phospho-Janus Kinase-2
**JAK2**	Janus Kinase-2
**P-STAT3**	Phospho-signal transducer and activator of transcription-3
**STAT3**	Signal transducer and activator of transcription-3
